# Malignant Odontogenic Tumors: A Clinicopathological Study of 53 Cases in Brazil

**DOI:** 10.1007/s12105-026-01952-w

**Published:** 2026-07-21

**Authors:** Lorena Paula de Paula, Flávia Sirotheau Correa Pontes, Felipe Paiva Fonseca, Pablo Agustin Vargas, Marcio Ajudarte Lopes, Alan Roger dos Santos Silva, Jacks Jorge Júnior, Paulo Victor Mendes Penafort, Adalberto Mosqueda-Taylor, Paulo Sérgio da Silva Santos, Vanessa Soares Lara, Cléverson Teixeira Soares, Décio dos Santos Pinto Júnior, Ramon Ribeiro, Elismauro Francisco de Mendonça, Diego Antônio Costa Arantes, Eneida Franco Vencio, Izadora Fernanda Veiga de Jesus Costa, Flávia Caló de Aquino Xavier, Jean Nunes dos Santos, Rogério Gondak, Filipe Modolo Siqueira, Juliana Lucena Schussel, Heliton Gustavo de Lima, Ciro Dantas Soares, Hélder Antônio Rebelo Pontes

**Affiliations:** 1 Oral Diagnosis Department, Piracicaba Dental School, University of Campinas (UNICAMP), Piracicaba, São Paulo, Brazil; 2 Service of Oral Pathology, João de Barros, Barreto University Hospital, Federal University of Pará, Belém, Pará, Brazil; 3 Department of Oral Surgery and Pathology, School of Dentistry, Federal University of Minas Gerais (UFMG), Belo Horizonte, Minas Gerais, Brazil; 4 Health Care Department, Universidad Autónoma Metropolitana, Mexico City, Mexico; 5 Bauru School of Dentistry of University of São Paulo, Bauru, SP Brazil; 6 Lauro de Souza Lima Institute, Bauru, SP Brazil; 7 Department of Stomatology, University of São Paulo, São Paulo, SP Brazil; 8 Department of Stomatologic Sciences, School of Dentistry, Federal University of Goiás, Goiânia, Brazil; 9 Department of Oral Pathology, School of Dentistry, Federal University of Bahia, Salvador, Brazil; 10 Department of Pathology, Federal University of Santa Catarina, Florianópolis, Santa Catarina, Brazil; 11 Department of Stomatology, Federal University of Paraná, Curitiba, PR Brazil; 12 Department of Dentistry, Federal University of Rio Grande Do Norte, Natal, Rio Grande Do Norte, Brazil

**Keywords:** Odontogenic tumors, Malignant odontogenic tumor, Odontogenic carcinoma, Ameloblastic carcinoma

## Abstract

**Objectives:**

This study aimed to investigate the clinicopathological features of malignant odontogenic tumors (MOTs) and to provide a comprehensive characterization that enhances the current understanding of this disease group.

**Methods:**

MOTs were retrieved from six Brazilian oral pathology services between 2007 and 2025. Demographic, clinical and radiographic data were collected from the charts, and the histological H&E-stained sections were revised by two blinded pathologists; previously available immunohistochemical and molecular findings were retrieved and reviewed in selected cases and the diagnosis was established according to the 2022 World Health Organization classification.

**Results:**

A total of 53 malignant odontogenic tumors were identified, predominantly affecting the mandible (83%), with a slight male predominance (male-to-female ratio, 1.12:1) and a mean age of 41.4 years. Ameloblastic carcinoma was the most frequent subtype (58.5%), followed by clear cell odontogenic carcinoma (18.9%), ameloblastic fibrosarcoma (13.2%), primary intraosseous carcinoma (7.5%), and ghost cell odontogenic carcinoma (1.9%).

**Conclusions:**

This multicenter review summarizes the main features of malignant odontogenic tumors. Although rare, these tumors often exhibit locally aggressive behavior, highlighting the need for early diagnosis, accurate histopathological evaluation, and standardized reporting with long-term follow-up.

## Introduction

Malignant odontogenic tumors (MOTs) are rare neoplasms of odontogenic origin, representing a small but clinically significant subset of maxillofacial pathologies. According to the latest World Health Organization (WHO) Classification of Head and Neck Tumours (5^th^ ed.), these lesions include entities such as sclerosing odontogenic carcinoma (SOC), ameloblastic carcinoma (AC), clear cell odontogenic carcinoma (COdC), ghost cell odontogenic carcinoma (GCOC), primary intraosseous carcinoma NOS (PIOC), odontogenic carcinosarcoma (OCS) and odontogenic sarcomas (OSs), all characterized by aggressive clinical behavior and significant diagnostic difficulties [1]. Despite their uncommon occurrence, MOTs pose considerable challenges for clinicians and pathologists, mainly due to their heterogeneity, overlapping features with benign odontogenic lesions, and the scarcity of large-scale studies that can inform evidence-based management [2, 3].

The diagnosis of MOTs requires careful integration of clinical, radiographic, and histopathological findings. Advances in immunohistochemistry and molecular biology have contributed to refining diagnostic criteria [4], including mutations in oncogenic signaling pathways such as BRAF p.V600E in AC, as well as epigenetic modifications [5], opening new avenues for understanding tumor biology and identifying potential therapeutic targets [2, 3].

Case series studies contribute significantly to understanding the clinical aspects of these neoplasms, including their anatomical distribution, sex predilection, age range, imaging characteristics, and post-treatment behavior. This multicenter case series, conducted across six oral pathology diagnostic centers, aims to enrich the existing literature by providing additional data on this uncommon group of malignant neoplasms affecting the gnathic bones.

## Methods

The present retrospective cross-sectional study included cases of MOTs retrieved from the archives of 6 oral pathology diagnostic services in Brazil: The University of São Paulo, the Federal University of Bahia, the João de Barros Barreto University Hospital, the Federal University of Rio Grande do Norte, the Federal University of Goiás and the University of Campinas.

Demographic, clinical, and radiographic data were obtained from laboratory archives for the period 2007—2025. The exclusion criteria encompassed cases in which previously documented data were irretrievable owing to incomplete medical records, specimens exhibited inadequate quality to permit definitive diagnostic verification, or patients withheld informed consent for data acquisition and participation in the investigation. The stages of data analysis, including laboratory procedures, were conducted at the Oral Pathology Division of the School of Dentistry at the University of Campinas (UNICAMP), in Piracicaba, São Paulo, Brazil. Data extraction was performed using a standardized form specifically designed in Microsoft Excel® software. Qualitative and quantitative variables were subsequently organized and descriptively analyzed using the Statistical Package for Social Sciences (SPSS) software, version 22.0 (SPSS Inc.). Paraffin-embedded blocks were sectioned at 3 µm, stained with hematoxylin and eosin (H&E), and independently assessed by two blinded pathologists under light microscopy. Cases showing diagnostic disagreement or interpretative uncertainty were jointly reassessed until consensus was reached, considering, when available, immunohistochemical, molecular, and clinical findings. Diagnostic reproducibility between observers, as well as agreement between the initial diagnosis and the final consensus diagnosis, was assessed using Cohen’s kappa coefficient. Histological classification followed the 2022 World Health Organization (WHO) criteria.

For selected cases, previously available immunohistochemical and molecular findings were retrieved from the original diagnostic records and reviewed according to their diagnostic relevance; no additional immunohistochemical or molecular analyses were performed for the present retrospective study.

This study was conducted in accordance with the tenets of the Helsinki Declaration for studies involving human subjects. It was approved by the local research ethics committee (FOP UNICAMP, process no. 89125625.7.0000.5418). Informed consent was obtained from all individual participants included in the study.

## Results

A total of 53 MOT cases were retrieved from six Brazilian institutions: 19 from the University of São Paulo, 13 from the University of Campinas, 7 from the João de Barros Barreto University Hospital, 6 from the Federal University of Bahia, 5 from the Federal University of Rio Grande do Norte, and 3 from the Federal University of Goiás. Cohen’s kappa analysis was performed to assess diagnostic agreement, demonstrating substantial interobserver agreement (κ = 0.63; 95% CI 0.43–0.80) and excellent agreement between the original diagnosis and the final consensus diagnosis (Cohen’s κ = 0.88; 95% CI 0.76–0.99). Among the 53 cases, 31 (58.5%) were diagnosed as AC, 10 (18.9%) as COdC, 7 (13.2%) as ameloblastic fibrosarcoma (AFS), 4 (7.5%) as PIOC, and 1 (1.9%) as GCOC. The demographic, clinical, and radiographic features of all retrieved MOT cases are summarized in *Online Resource 1*.

Regarding sex distribution, 28 patients (52.8%) were male and 25 (47.2%) were female, yielding a male-to-female ratio of 1.12:1. Patient age ranged from 12 to 89 years, with a mean age of 41.4 years. The mean age was 42.5 years among female patients and 40.4 years among male patients.

**Fig. 1:**
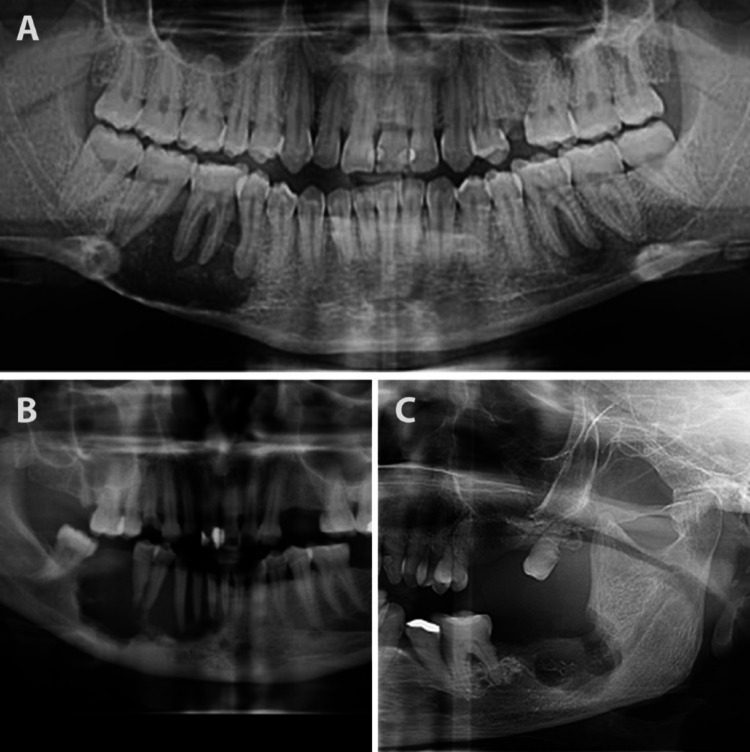
Radiographic features of malignant odontogenic tumors (MOTs) **a** Ghost cell odontogenic carcinoma. Unilocular lesion in the mandibular molar region (case 05) **b** Ameloblastic carcinoma. Unilocular radiolucent lesion involving both the anterior and posterior regions of the mandible (case 20). **c** Primary intraosseous carcinoma, NOS. Multilocular radiolucent lesion with scalloped margins located in the posterior mandibular region (case 02)

Anatomically, most lesions involved the mandible (44 cases; 83.0%), followed by the maxilla (7 cases; 13.2%) and the maxillary sinus (1 case; 1.9%); in one case (1.9%), the anatomical site was not specified. Among mandibular lesions, 18 cases (40.9%) involved the posterior region, 7 (15.9%) the anterior region, 6 (13.6%) both the anterior and posterior regions, and 2 (4.5%) the condylar region. In 11 mandibular cases (25%), the specific mandibular site was not reported. Among maxillary lesions, 4 cases (57.1%) were located in the posterior region, 1 (14.3%) in the anterior region, and 2 (28.6%) involved both anterior and posterior regions. One additional case (1.9%) occurred in the maxillary sinus. Radiographically, the majority of lesions presented a radiolucent pattern (n = 19; 35.8%) (Fig. 1), whereas a mixed radiolucent–radiopaque appearance was infrequently observed (n = 1; 1.9%). With respect to internal architecture, multilocular lesions predominated (n = 12; 22.6%) over unilocular configurations (n = 6; 11.3%). Root resorption was identified in a small subset of cases (n = 2; 3.8%), while tooth displacement was documented in four cases (7.5%). Notably, radiographic data were unavailable in 25 cases (47.2%). Computed tomography (CT) data were available in five cases, all of which demonstrated hypodense lesions.

The most frequent clinical feature was swelling, observed in 8 cases (15.1%). Mucosal erythema was observed in 4 cases (7.5%), and ulceration of the overlying mucosa was observed in 4 cases (7.5%). Pain was reported in 4 patients (7.5%). Areas of surface necrosis were observed in 3 cases (5.7%), and paresthesia was recorded in 2 patients (3.8%). In addition, tooth mobility and dysphagia were documented in one case each (1.9%), reflecting the extent of alveolar bone destruction. Table 1 summarizes the main clinicopathological features of each MOT.

**Table 1:** Clinicopathological characteristics of 53 malignant odontogenic tumors included in the study

Variables	AC	COdC	AFS	PIOC	GCOC
Cases n (%)	31(58.5)	10 (18.9)	7 (13.2)	4 (7.5)	1 (1.9)
Age, mean (min–max)	40.5 (14–74)	48.1 (21–89)	25.4 (12–72)	63 (47–73)	28
Sex male/female	19/12	5/5	3/4	1/3	0/1
Signs and symptoms n (%)	Swelling 5 (16.1); Erythema 3 (9.7); Ulceration 3 (9.7); Pain 2 (6.5); Necrosis 2 (6.5); Paresthesia 1 (3.2); Tooth mobility 1 (3.2); Dysphagia 1 (3.2); NR 23 (74.2)	NR 10 (100.0)	Swelling 1 (14.3); Erythema 1 (14.3); Pain 1 (14.3); Ulceration 1 (14.3); Necrosis 1 (14.3); NR 6 (85.7)	Swelling 2 (50.0); Pain 1 (25.0); Paresthesia 1 (25.0); NR 1 (25.0)	NR 1 (100.0)
Radiographic aspect n (%)	Radiolucent 11 (35.5); Multilocular 9 (29.0); Unilocular 5 (16.1); Mixed 1 (3.2); Tooth displacement 1 (3.2); Root resorption 1 (3.2); NR 12 (38.7)	Radiolucent 4 (40.0); Multilocular 1 (10.0); Tooth displacement 1 (10.0); Root resorption 1(10.0); NR 6 (60.0)	Radiolucent 2 (28.6); Multilocular 1 (14.3); Tooth displacement 2 (28.6); NR 4 (57.1)	Radiolucent; multilocular 1 (25.0); NR 3 (75.0)	Radiolucent and unilocular 1 (100.0)
Site n (%)	Mandible, UR 10 (32.3); Condyle 1 (3.2); Anterior mandible 4 (12.9); Mandibular molars 5 (16.1); Anterior, PM, and molar regions of the mandible 5 (16.1); Anterior and PM regions of the mandible 1 (3.2); Maxillary molars 1 (3.2); Maxillary PM and molars 2 (6.5); Maxillary sinus 1 (3.2); NR 1 (3.2)	Mandible, UR 1 (10.0); Anterior mandible 1 (10.0); Mandibular PM 1 (10.0); Mandibular molars 4 (40.0); Anterior, PM, and molar regions of the maxilla 2 (20.0); Maxillary molars 1 (10.0)	Condyle 1 (14.3); Anterior mandible 1 (14.3); Mandibular molars 4 (57.1); Mandibular PM and molar regions 1 (14.3)	Mandibular molars 2 (50.0); Anterior mandible 1 (25.0); Anterior maxilla 1 (25.0)	Mandibular PM and molars 1 (100.0)
Treatment n (%)	Surgery 4 (12.9); None 1 (3.2); NR 26 (83.9)	NR 10 (100.0)	Surgery + CT + RT 1 (14.3); NR 6 (85.7)	Surgery 1 (25.0); CT + RT 1 (25.0); NR 2 (50.0)	NR 1 (100.0)
Survival n (%)	Alive 4 (12.9); Dead 1 (3.2); Alive (recurrence) 1 (3.2); NR 25 (80.6)	NR 10 (100.0)	NR 7 (100.0)	Alive 2 (50); Dead (metastasis) 1 (25.0); NR 1 (25.0)	Alive (recurrence) 1 (100.0)
Follow-up period, months, mean (min–max)	44.8 (24–84)	NR	NR	33.3 (12–60)	11

Abbreviations: AC: Ameloblastic carcinoma; AFS: Ameloblastic fibrosarcoma; COdC: Clear cell odontogenic carcinoma; CT: Chemotherapy; GCOC: Ghost cell odontogenic carcinoma; NR: Not reported; PIOC: Primary intraosseous carcinoma, NOS; PM: Premolars; RT: Radiotherapy; UR: Unspecified region

**Fig. 2:**
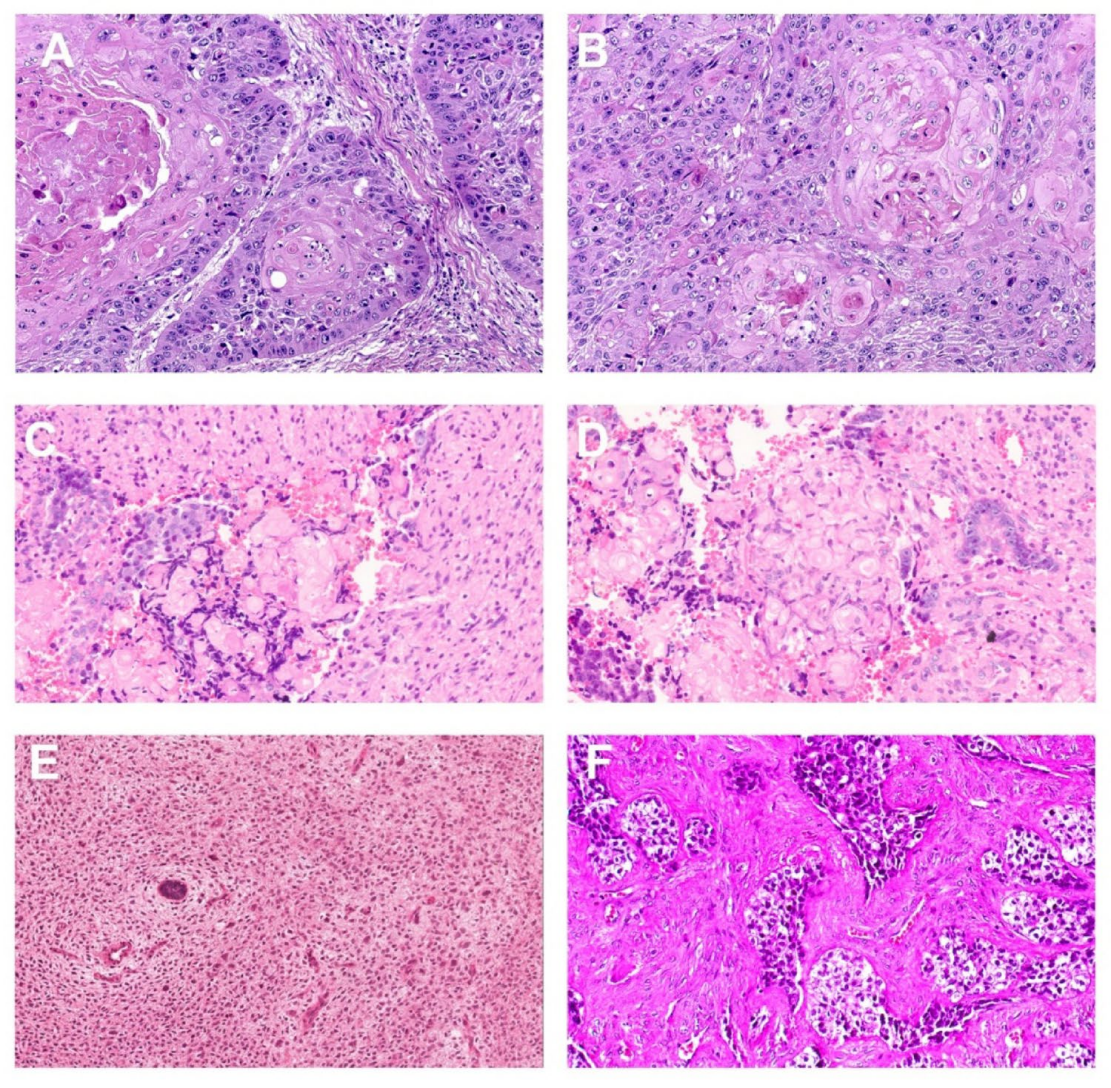
Histopathological findings **a–b** Ameloblastic carcinoma (case 20) **c–d** Ghost cell odontogenic carcinoma (case 05) **e** Ameloblastic fibrosarcoma (case 23) **f** Clear cell odontogenic carcinoma (case 34)

Microscopic features observed in 4 of the 53 cases are illustrated in Fig. 2. Immunohistochemical results were available in twenty-two cases, as summarized in Table 2. In 3 cases of AC (Cases 1, 3, and 4), molecular testing by pyrosequencing revealed the *BRAF p.V600E* mutation. In the sample from Case 5, diagnosed as GCOC, next-generation sequencing (NGS) revealed a *CTNNB1* mutation and *PTEN* loss, whereas in Case 31, diagnosed as COdC, an *EWSR1* rearrangement was detected by fluorescence in situ hybridization (FISH) (Table 3).

**Table 2:** Antibodies used for immunohistochemical evaluation of MOTs

Case	Diagnosis	Immunohistochemical
01	Ameloblastic carcinoma	CK19 + , Ki67 + , CK14 + , CK5/6-, p53 +
03	Ameloblastic carcinoma	CK19 + , Ki67 + , CK14 + , CK5/6 + , CK20-, p16-, p53 + , CK7-
04	Ameloblastic carcinoma	CK19 + , Ki67 + , CK14 + , CK5/6 + , p53 +
08	Ameloblastic carcinoma	Ki67 + , AE1/AE3 + , Vimentin + , p16 +
19	Ameloblastic carcinoma (squamous differentiation)	Ki67 + , AE1/AE3 + , CK5 + , CK7 + , CK8 + , CK14 + , CK18 + , CK19 + , SMA + , S100 + , p53 +
19	Ameloblastic carcinoma (undifferentiated areas)	Ki67 + , AE1/AE3 + , CK5 + , CK7 + , CK8 + , CK14 + , CK18 + , CK19 + , SMA + , S100 + , p53 +
24	Ameloblastic carcinoma	Ki67 +
27	Ameloblastic carcinoma	Ki67 +
29	Ameloblastic carcinoma	Ki67 +
33	Ameloblastic carcinoma	Ki67 +
45	Ameloblastic carcinoma	Ki67 +
49	Ameloblastic carcinoma	CK14 + , CK19 + , SMA + , BCL2 + , Ki67 +
18	Ameloblastic fibrosarcoma	Ki67 + , PCNA + , Bcl-2 +
23	Ameloblastic fibrosarcoma	Ki67 +
30	Ameloblastic fibrosarcoma	Ki67 +
37	Ameloblastic fibrosarcoma	Ki67 +
02	Primary intraosseous carcinoma, NOS	CK5/6 + , CK19 + , Ki67 + , p53-, p16-
09	Primary intraosseous carcinoma, NOS	Ki67 + , AE1/AE3, Vimentin + , p16 + , LCA-, HMB-45-, CK7-, CK20-, CK19-, IA-4-, 34βE12-, Desmin-
53	Primary intraosseous carcinoma, NOS	Ki67 + , p53 +
31	Clear cell odontogenic carcinoma	CK5 + , CK14 + , EMA + , p63 + ; CK19 + , CK7 + , CK20-, Calponin-, SMA-
32	Clear cell odontogenic carcinoma	Ki67 +
34	Clear cell odontogenic carcinoma	AE1/AE3 + and CK5 + ; p40 + , p63 + EMA + ; Ki67 + ; CK7-, CK14-, LCA-, CK20-, CD10-, RCC-, SOX10-, S100-, SYP-, CgA-, Vimentin-, CD99-
05	Ghost cell odontogenic carcinoma	Beta-catenin + , CK19 + . Ki67 + , CK7 + , p53 +

**Table 3:** Molecular findings of MOTs

Case	Diagnosis	Molecular analysis	Method
01	Ameloblastic carcinoma	BRAF p.V600E	pyrosequencing
03	Ameloblastic carcinoma	BRAF p.V600E	pyrosequencing
04	Ameloblastic carcinoma	BRAF p.V600E	pyrosequencing
05	Ghost cell odontogenic carcinoma	CTNNB1 (mutation), PTEN (loss)	NGS
31	Clear cell odontogenic carcinoma	EWSR1	FISH

Abbreviations: NGS: Next-Generation Sequencing; FISH: Fluorescence In Situ Hybridization

Regarding therapeutic management, 8 patients had detailed treatment information available. 5 cases (9.4%) were managed exclusively with surgical excision, 1 case (1.9%) received combined surgery, chemotherapy, and radiotherapy, 1 case (1.9%) underwent chemoradiotherapy without surgery, and 1 case (1.9%) received only palliative care due to advanced disease stage (case 19). Nevertheless, treatment information was not reported for the vast majority of cases (n = 45; 84.9%).

In terms of clinical outcomes, follow-up data were available for 10 cases (18.9%). Among these, 8 patients remained alive (15.1%), of whom 2 developed tumor recurrence (25.0% of surviving cases; 3.8% of the total cohort), whereas 6 showed no evidence of recurrence (75.0% of surviving cases; 11.3% of the total cohort). Two patients (3.8%) died of disease, and metastatic disease was identified in one of these cases (1.9%). Clinical outcome data were not reported in most cases (n = 43; 81.1%). The mean follow-up period was 37.2 months, ranging from 11 to 84 months.

**Fig. 3:**
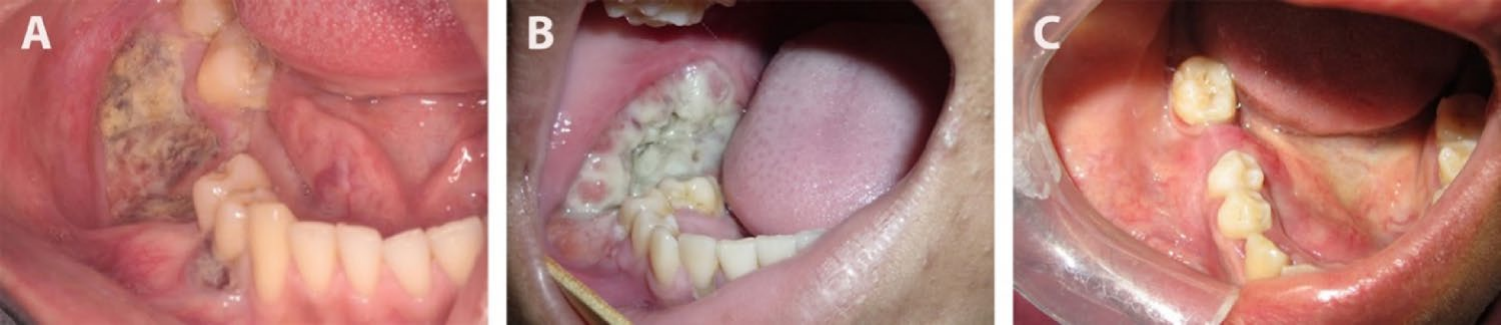
Clinical features of ameloblastic carcinoma **a** An ulcerated lesion extending from the premolar to the molar region of the mandible, with areas of necrosis (case 16) **b** Mandibular swelling with an extensive area of necrosis (case 21) **c** Swelling in an edentulous mandibular region between the premolar and molar areas, with surrounding erythematous mucosa (case 20)

As the most prevalent lesion, AC accounted for 31 cases, representing 58.5% of all cases in this study. Among the affected patients, 19 (61.3%) were male and 12 (38.7%) were female, yielding a male-to-female ratio of 1.58:1. The mean age of the patients was 40.5 years. The average age among male patients was 43 years, while female patients presented a mean age of 36.7 years. Representative clinical features of AC are shown in Fig. 3. The clinicopathological features of AC are summarized in Table 1. In 2 patients (cases 17 and 19), the development of AC was preceded by a pre-existing ameloblastoma, suggesting malignant transformation. Three of the 5 surviving patients were treated surgically, whereas the patient who died received only palliative care due to the severity of the clinical condition.

The second most prevalent lesion was COdC, accounting for 10 cases. Of these, 5 (50.0%) occurred in female patients and 5 (50.0%) in male patients, resulting in a male-to-female ratio of 1:1. The mean age was 48.1 years, ranging from 21 to 89 years. The mean age among female patients was 51 years, whereas it was 45.2 years among male patients. Radiographic and anatomical information of COdC is available in Table 1; however, clinical signs and symptoms were not reported for these cases. Information regarding treatment and follow-up was not available.

AFS accounted for 7 cases. The mean age was 25.4 years, ranging from 12 to 72 years. Four patients were female (57.1%) and three were male (42.9%), resulting in a male-to-female ratio of 0.75:1. The mean age among female patients was 33.7 years, whereas among male patients it was 14.3 years. All seven cases involved the mandible, including five in the posterior region, one in the anterior region, and one in the mandibular condyle. Only one case included a description of clinical features, comprising pain, necrosis, ulceration, mucosal erythema, and swelling. This same case was the only one in which treatment was reported, consisting of surgery combined with chemotherapy and radiotherapy. One case exhibited a bilateral lesion pattern. No follow-up information was reported in any of the cases.

Four patients were diagnosed with PIOC. The mean age was 63 years, ranging from 47 to 73 years. Three patients were female (75.0%) and one was male (25.0%). The mean age of the female patients was 68.3 years, whereas the single male patient was 47 years old. Treatment was reported in two cases: one patient underwent chemotherapy combined with radiotherapy, and another was treated surgically. Both patients showed favorable survival outcomes and remained alive and disease-free at 60 and 12 months, respectively. One patient succumbed to the disease due to metastasis 28 months after diagnosis. Information regarding the treatment performed in this case was not available. In two cases, PIOC arose from a previously diagnosed odontogenic keratocyst and in one case the malignant lesion arose from a residual cyst.

In the single case of GCOC, the patient was a 28 year-old female. The lesion was located in the posterior mandible and exhibited a unilocular appearance on panoramic radiography. Recurrence was observed during an 11-month follow-up period.

## Discussion

MOTs are rare entities, accounting for approximately 2% of all odontogenic neoplasms. They typically exhibit clinically aggressive behavior, characterized by a high propensity for local recurrence, bone infiltration, and, in some cases, metastatic potential [3]. According to the literature, the mean age at presentation for these lesions ranges from the fourth and to the sixth decades of life [3, 6]. Similarly, the present study demonstrated a comparable age pattern, with a mean age of 41.4 years. Overall, the literature indicates a male predominance among patients with malignant odontogenic tumors [2, 6], and similarly, the present study demonstrated a slight male predilection. The mean age for female patients was 42.5 years, whereas for male patients it was 40.4 years, indicating that these tumors tend to occur slightly earlier in men.

The most frequently reported clinical features in the literature include swelling and pain, which were also predominant findings in our study [3, 7]. Ulcerations have also been described in previous reports on these lesions and, although not commonly observed, they were notable findings in the present series [6]. Three cases with mucosal necrosis were observed. Compared with previously reported cases, mucosal necrosis is not a frequent clinical feature of MOTs.

Consistent with the literature, a strong association between MOTs and the posterior region of the mandible was also seen in our study [9]. This preferential localization may be explained by the greater activity of odontogenic epithelial remnants in the posterior mandible, as well as by the higher frequency of some locally aggressive lesions in this region that are susceptible to malignant transformation [10].

AC is a primary and rare malignant odontogenic tumor defined by the coexistence of typical histopathological features of an ameloblastoma and cytological evidence of malignancy, including nuclear pleomorphism, hyperchromatism, increased mitotic activity, and cellular atypia [1, 17]. According to the literature, AC is reported as the most frequent entity among the MOTs, accounting for approximately 30% of all cases in this group [1]. Similarly, in the present study, AC was the most prevalent lesion, accounting for 58.5% of all MOTs analyzed. As in previous reports, most cases in our series were located in the posterior region of the mandible [8, 11]. Maxillary involvement, particularly within the maxillary sinus, is considered uncommon and may pose diagnostic and surgical challenges due to the area’s anatomical complexity. In our study, we reported one case involving the maxillary sinus region, consistent with the rarity of this location for AC described in the literature.

There is some disagreement in the literature regarding the sex predilection of this lesion. Some studies have suggested a female predominance [11], whereas others have reported a male predominance [12, 13–15]. In the present study, a male predominance was observed, and female patients developed the disease at an earlier age compared with male patients. Surgical excision with tumor-free margins remains the gold standard for the treatment of AC, as wide local resection significantly reduces recurrence and improves overall survival. Adjuvant radiotherapy may be considered in selected cases, particularly in elderly patients or those who are medically unfit for extensive surgical procedures, as well as in the presence of high-risk pathological features such as positive or close surgical margins, perineural invasion, or regional metastasis. In addition, patients harboring documented BRAF mutations may benefit from molecularly targeted therapeutic approaches, including BRAF inhibitor therapy [11, 16]. Among surviving AC patients with available treatment information, three were treated surgically.

BRAF p.V600E mutations are predominantly detected in mandibular ameloblastomas and AC, being associated with increased local aggressiveness, higher recurrence rates and poorer prognosis [16, 17]. In our series, an association between AC and the BRAF p.V600E mutation was identified, with two cases in the posterior mandible and one in the posterior maxilla.

Ki67, a marker of cellular proliferation and growth, shows higher expression levels in AC than in conventional ameloblastoma, serving as a reliable indicator of increased growth potential and biological aggressiveness, as well as a dependable marker for distinguishing between these two entities [18, 20]. However, Penafort et al. (2024) reported that this marker does not consistently exhibit uniform immunoreactivity, which may result from interpretative discrepancies, thereby limiting its reliability as a sole diagnostic discriminator between these lesions [19].

The histopathological diagnosis of malignant odontogenic tumors remains particularly challenging because of their rarity, heterogeneous morphology, and frequent overlap with benign odontogenic lesions and non-odontogenic malignancies [1–3, 27]. In several cases, malignant transformation may occur in association with pre-existing odontogenic lesions, including ameloblastoma, odontogenic keratocyst, and other odontogenic cysts, further complicating the distinction between benign lesions with atypical features and true odontogenic malignancies [2, 3, 27, 28]. This diagnostic difficulty is particularly evident in clear cell odontogenic carcinoma, whose microscopic features may overlap with other clear cell–containing tumors of the jaws, including gland neoplasms, metastatic tumors, melanocytic lesions, and metastatic tumors, as renal cell carcinoma [1, 21–23]. Therefore, diagnosis should not rely exclusively on morphology, but should integrate clinical presentation, anatomical location, radiographic findings, histopathological features, and, when available, immunohistochemical and molecular data [1, 3, 4, 22, 27]. In this context, immunohistochemical profiling and molecular alterations, such as EWSR1 rearrangements in clear cell odontogenic carcinoma, may provide important diagnostic support when interpreted within the appropriate clinicopathological and immunohistochemical context and may help exclude histological mimics [1, 21–23]. Nevertheless, given the limited number of well-documented cases and the lack of standardized ancillary testing across retrospective series, the differential diagnosis of malignant odontogenic tumors continues to require careful clinicopathological correlation and expert pathological review.

Clear cell odontogenic carcinoma was the second most frequent tumor in our series. It is an uncommon and aggressive neoplasm and, to the best of our knowledge, this study represents the first case series to document such a number of cases within a single cohort (n = 10) [21]. According to the literature, this tumor predominantly affects women; however, in our study, no sex predilection was observed, and the mean age of the female patients was 51 years, which is comparable to the mean age reported for malignant odontogenic tumors overall. Regarding anatomical distribution, our findings are consistent with previous reports, with a higher predilection for the posterior region [21–23].

The expression patterns of CK14, CK19, EMA, p40, and p63 may support the diagnosis of COdC. Conversely, the absence of selected markers associated with metastatic renal cell carcinoma, melanocytic tumors, or salivary gland neoplasms may assist in excluding other clear cell–containing malignancies. [22]. Furthermore, integrating immunohistochemical analysis with EWSR1 gene rearrangements has been shown to enhance diagnostic accuracy. Previous studies have demonstrated that EWSR1 gene fusions, most commonly involving ATF1, are frequently identified in COdC, thereby providing a valuable molecular basis for diagnosis [21, 22].

With 7 cases, ameloblastic fibrosarcoma (AFS) represented the third most prevalent malignant odontogenic tumor in the present review. AFS is a rare neoplasm that is thought to arise in a large number of the reported cases from dysplastic transformation of the mesenchymal component in its precursor lesion, the ameloblastic fibroma (AF) [24, 25]. Previous studies have reported a male predominance, whereas the present series demonstrated a slight female predilection [24]. Consistent with the literature, AFS in the present study predominantly affected younger patients, with a mean age of 25.4 years and a wide age range (12–72 years) [25]. This age distribution supports the concept that AFS tends to occur earlier in life compared with other malignant odontogenic tumors.

Given the ameloblastic morphology, the BRAF V600E point mutation has been identified in the majority of reported AFS cases. Furthermore, comprehensive genomic profiling of patients with AFS has revealed EGFR exon 20 insertions and MDM2 amplification, which have emerged as potential molecular drivers of AFS development and progression [25, 26].

As the fourth most prevalent lesion in our series, PIOC accounted for 4 reported cases. According to Jain et al. (2025), only about 60 cases have been described in the literature to date, highlighting the extreme rarity of this malignancy [27]. Due to its uncommon occurrence, the etiopathogenesis and biological behavior of this tumor remain poorly understood. In the latest World Health Organization (WHO) Classification of Head and Neck Tumours (5th edition, 2022), this entity is defined as a central jaw carcinoma that cannot be categorized as any other specific type of odontogenic carcinoma [1]. The etiology of this lesion appears to be related to the proliferation of odontogenic epithelial remnants and the epithelial lining of odontogenic cysts, with chronic inflammation acting as a predisposing factor [14, 29, 30]. Approximately 60% of reported cases of this tumor have been associated with previous residual cysts, while about 40% are linked to dentigerous cysts and odontogenic keratocysts [28]. This reinforces the hypothesis that malignant transformation may occur secondary to long-standing cystic lesions, particularly in the presence of persistent inflammatory stimuli [27].

Among the benign lesions most frequently associated with malignant transformation are odontogenic keratocysts and residual cysts. In the present study, one case was preceded by a residual cyst and two cases by an odontogenic keratocyst, supporting the hypothesis that malignant transformation may arise from pre-existing odontogenic cystic lesions. In this series, three patients were female and one was male. The lesions predominantly involved the mandible, with two cases located in the posterior region and one in the anterior region, while a single lesion affected the anterior maxilla. These findings contrast with the male predominance reported in previous studies [28, 29].

Cervical and distant metastasis rates for PIOC have been reported to be approximately 10.3% each, according to previous reviews [29]. These relatively low rates may be attributed to the limited number of well-documented cases in the literature, which limits an accurate assessment of the tumor’s metastatic potential. In our study, one patient died due to metastatic disease, reinforcing the malignant nature and potential aggressiveness of PIOC.

The immunohistochemical profile for diagnosing PIOC is not yet fully understood in the literature. However, it has been suggested that p63, CK19, and Ki67 may assist in distinguishing PIOC from other histologically comparable odontogenic and non-odontogenic malignancies [27, 28]. In our study, a high Ki67 labeling index was observed, indicating increased proliferative activity. p53 was strongly positive, while CK19 showed weak, diffuse expression, findings consistent with previously reported literature.

Ghost cell odontogenic carcinoma (GCOC) is an exceptionally rare malignant odontogenic tumor, accounting for approximately 0.23% of all odontogenic tumors and classically defined by the presence of ghost cells and, in some cases, dentinoid deposition [1, 33, 34]. Epidemiologically, most published series describe a male predominance and a predilection for the anterior maxilla [31, 32]. Against this background, our case—occurring in a 28-year-old female with a posterior mandibular lesion—represents an uncommon demographic and topographic presentation that broadens the clinicopathologic spectrum of GCOC.

Prior studies have shown overexpression of Ki67 and p53 in ghost cell odontogenic carcinoma (GCOC), indicating a high proliferative fraction and dysregulated cell-cycle control [35]. Our case mirrored these findings, supporting the tumor’s aggressive behavior. Molecular testing further refines diagnosis of ghost-cell–rich odontogenic lesions by revealing activating CTNNB1 mutations that stabilize β-catenin, drive nuclear accumulation, and induce transcription of pro-proliferative targets and aberrant differentiation. Such alterations are frequent in GCOC and occur in a subset of calcifying odontogenic cysts, underscoring a shared Wnt/β-catenin axis [36]. In our patient, we detected a CTNNB1 mutation with nuclear β-catenin immunoreactivity, consistent with pathway activation. Moreover, loss or mutation of the tumor suppressor PTEN—one of the most commonly altered genes in human cancers—promotes cell survival, proliferation, and invasiveness [37].

This study has some limitations, including the small number of cases, reflecting the rarity of malignant odontogenic tumors, and limited clinical follow-up for a subset of patients, as most participating centers are referral units dedicated primarily to oral diagnostic services. In addition, methodological heterogeneity, particularly regarding immunohistochemical and molecular analyses, represents an inherent limitation of the retrospective study design. Nevertheless, these limitations underscore the need for multicenter studies with standardized protocols and comprehensive molecular profiling to further elucidate the biological behavior and prognostic factors of malignant odontogenic tumors.

In conclusion, MOTs constitute a rare group of neoplasms with a slight male predilection that predominantly affect the posterior mandible. These tumors typically present as multilocular radiolucent findings on imaging examinations and may be associated with root resorption and tooth displacement. Despite their low incidence, MOTs exhibit aggressive biological behavior, with a recognized potential for local recurrence and metastatic spread. Surgical resection remains the cornerstone of treatment, but the extent of surgery, role of adjuvant therapies, and long-term prognostic factors remain subjects of debate.

## Data Availability

All data supporting the findings of this study are available within the paper and its Supplementary Information.
